# RSV Vaccines: Targeting Prefusion F and G Proteins from Structural Design to Clinical Application

**DOI:** 10.3390/vaccines13111133

**Published:** 2025-11-03

**Authors:** Dongrunhan Yu, Chengwei Zhang, Yunyi Qi, Ziyi Liu, Di Yang, Nan Zhao, Zunhui Ke, Xiaoxia Lu, Yan Li

**Affiliations:** 1 Department of Pathogen Biology, School of Basic Medicine, Tongji Medical College and State Key Laboratory for Diagnosis and Treatment of Severe Zoonotic Infectious Diseases, Huazhong University of Science and Technology, 13 Hangkong Road, Wuhan 430030, China; u202113277@hust.edu.cn (D.Y.); d202481972@hust.edu.cn (D.Y.); 2 Union Hospital, Tongji Medical College, Huazhong University of Science and Technology, Wuhan 430021, China; u202113647@hust.edu.cn; 3 Tongji Hospital, Tongji Medical College and State Key Laboratory for Diagnosis and Treatment of Severe Zoonotic Infectious Diseases, Huazhong University of Science and Technology, Wuhan 430030, China; u202113548@hust.edu.cn (Y.Q.); u202113649@hust.edu.cn (Z.L.); 4 Hubei Provincial Key Laboratory of Pediatric Genetic Metabolic and Endocrine Rare Diseases, Wuhan 430030, China; 5 Department of Respiratory Medicine, Wuhan Children’s Hospital, Tongji Medical College, Huazhong University of Science and Technology, Wuhan 430014, China; nanzhao@whu.edu.cn; 6 Pediatric Respiratory Disease Laboratory, Institute of Maternal and Child Health, Wuhan Children’s Hospital, Tongji Medical College, Huazhong University of Science and Technology, Wuhan 430014, China; 7 Department of Blood Transfusion, Wuhan Children’s Hospital, Tongji Medical College, Huazhong University of Science and Technology, Wuhan 430014, China; zunhui.k@whu.edu.cn

**Keywords:** RSV, subunit vaccines, prefusion F protein, attachment glycoprotein, virus-like particle

## Abstract

**Background**: Respiratory syncytial virus (RSV) is a major pathogen of acute lower respiratory tract infection (LRTI) in infants, the elderly, and immunocompromised individuals. This review focuses on the progress of RSV vaccine development, especially subunit vaccines targeting the fusion protein (F) and attachment glycoprotein (G), aiming to summarize key strategies, challenges, and future directions in the field. **Methods**: The review is based on a comprehensive literature search and analysis of recent studies on RSV vaccine development, with a specific focus on subunit vaccines and related technologies. **Results**: Approved vaccines such as Abrysvo and Arexvy utilize structural engineering to stabilize the prefusion conformation of the F protein (PreF), thereby exposing neutralizing epitopes. Subunit vaccine candidates such as DS-Cav1 and DT-PreF enhance stability through disulfide bonds and dityrosine linkages, while ADV110 targets the conserved domain of the G protein to elicit cross-strain immunity. Virus-like particle (VLP) vaccines like IVX-A12 combine RSV and human metapneumovirus antigens to provide broad-spectrum immunity. However, challenges exist, including maintaining PreF stability, overcoming immunosenescence in the elderly, and addressing safety concerns like Guillain-Barré syndrome (GBS). **Conclusions**: Future RSV vaccine development should center on combined PreF-G protein vaccines, VLP technology, and optimizing cold-chain logistics to improve global accessibility and overcome existing challenges, thereby providing more effective prevention and control of RSV infections.

## 1. Introduction

As a highly contagious virus, RSV is one of the most common pathogens causing acute lower respiratory tract infection (LRTI), particularly in infants, the elderly, and immunocompromised individuals [1,2,3,4]. Globally, RSV affects approximately 64 million people and causes 160,000 deaths annually [5], with the impact being disproportionately severe in neonates and the elderly due to specific risk factors.

Newborns are particularly vulnerable to RSV infection due to several physiological and immunological factors. First, their immune system is underdeveloped with immature dendritic cells and low antiviral cytokine levels, which delay effective immune responses. Furthermore, infants are vulnerable to airway blockage resulting from RSV-induced inflammation due to their physical characteristics, such as weak respiratory muscles, soft tracheobronchial cartilage, and short airways. Additionally, while maternal antibodies offer some protection, they do not completely prevent infection.

The severity of RSV infection in elderly individuals is affected by a variety of factors. A key factor is immunosenescence, which impairs T and B cell function and weakens antiviral immune responses. Additionally, the presence of common comorbidities such as chronic obstructive pulmonary disease (COPD) and cardiovascular diseases can exacerbate the consequences of RSV infection. The aging respiratory system is characterized by reduced alveolar elasticity and impaired ciliary action.

**Figure 1:**
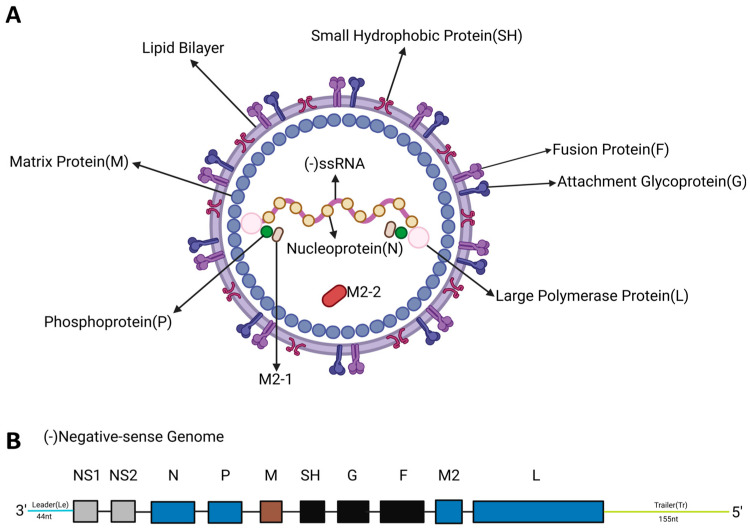
Structural and genomic organization of RSV. (**A**). Schematic diagram of RSV structure. The viral envelope is composed of glycoproteins such as F protein, G protein, and small hydrophobic protein (SH). The ribonucleoprotein complex of RSV is composed of RNA, nucleoprotein (N protein), the large polymerase protein (L), the phosphoprotein (P), and the processivity factor M2-1. The matrix protein (M) is located between the RNP and the envelope. (**B**). Schematic diagram of RSV genome structure. Eleven RSV proteins are encoded by ten genes on a negative-sense RNA. M2 has two slightly overlapping open reading frames encoding two proteins: M2-1 and M2-2.

RSV is classified under the order Mononegavirales, the family *Pneumoviridae*, and the genus *Orthopneumovirus*. This virus is a non-segmented negative-strand RNA virus. The genome of RSV has an approximate length of 15.2 kb and comprises 11 open reading frames, which encode 9 structural proteins (F, G, N, P, M, SH, L, M2-1, and M2-2) and 2 non-structural (NS) proteins (Figure 1) [6,7,8].

The F and G glycoproteins are both transmembrane proteins that are highly glycosylated and mediate viral entry. The F protein, which directs virus-cell and cell-cell fusion, exhibits strong genetic and antigenic conservation. It presents multiple neutralizing epitopes and can elicit potent neutralizing antibody responses, making it a primary vaccine target [9]. There is only a 25-amino acid difference in the extracellular domain of the F protein between the two subtypes A and B of RSV. Due to its high level of conservation among RSV strains, the F protein is the main vaccine target antigen [10].

In contrast, the G protein exhibits the highest variability of all the main proteins in RSV. Its sequence variation defines the RSV subtypes, with most of the variation concentrated in the mucin domain at the amino and carboxyl terminus of the protein [9]. However, G protein also contains a central conserved region that facilitates viral attachment by interacting with cell surface heparan sulfate receptor and contains a neutralization-sensitive epitope [9]. Furthermore, the G protein contains a central conserved domain (CCD) with a low degree of glycosylation and the presence of a CX3C motif. This CX3C motif (amino acid sequence CWAIC at positions 182–186 in the RSV A2 112 strain) is necessary for G protein to mimic the chemokine fractalkine and bind to the host CX3CR1 receptor [11].

In summary, the F and G proteins are suitable targets for neutralizing antibodies in RSV vaccine design due to their important roles and genetic conservation. This review provides a comprehensive overview of current RSV vaccines, with a focus on strategies targeting the F and G proteins based on their functional mechanisms.

## 2. Current Status of RSV Vaccine Research

Natural infection with RSV does not induce durable immunity, leading to frequent reinfections throughout life. Therefore, specific immunoprophylaxis is of great significance [12]. During the development of early RSV vaccines, enhanced respiratory disease (ERD) attracted extensive attention. ERD refers to the more severe disease occurring after natural infection in RSV-naïve infants who had been vaccinated. It was initially observed in relation to the formalin-inactivated RSV vaccine (FI-RSV) that was developed during the 1960s. This vaccine was prepared using the Bernett strain cultured in African green monkey kidney tissue, inactivated with formalin, and concentrated via alum precipitation. However, it failed to provide effective protection and, in some cases, even exacerbated disease severity. In 1967, two children died from ERD following FI-RSV vaccination. This incident seriously hindered the development of RSV vaccines for decades and prompted the Food and Drug Administration (FDA) to impose more stringent review criteria for RSV vaccines. ERD was partly attributed to the use of formaldehyde, which inactivates the virus but failed to keep the F protein in the PreF conformation or to induce effective neutralizing antibodies [13]. When these non-neutralizing antibodies subsequently encounter wild-type RSV, they may paradoxically enhance viral infection and replication, thus triggering a series of subsequent reactions and diseases [14,15]. Consequently, this requires more complex and cautious considerations in the research and development of RSV vaccines.

In the background of RSV infection and vaccination, antibody-dependent enhancement (ADE) is a potential mechanism of ERD. Antibodies against the virus may fail to neutralize it and instead enhance its infectivity. This phenomenon typically occurs when the antibody concentration is insufficient to achieve complete neutralization. These sub-neutralizing levels of antibodies can bind to the virus and form immune complexes. The complexes facilitate viral entry into immune cells via Fcγ receptor-mediated endocytosis, thereby increasing viral replication and infection. Furthermore, the immune complexes can also activate the complement system, triggering the release of inflammatory mediators and the recruitment of immune cells, which may aggravate the inflammatory response and tissue damage [14,15].

Numerous studies have shown that the non-neutralizing antibody response elicited by FI-RSV is closely associated with insufficient activation of the Toll-like receptor (TLR) signaling pathway during vaccination. This defect in TLR signaling directly affects the affinity maturation process of B cells, resulting in the production of antibodies with poor virus-neutralizing capacity [16]. In addition, at the cellular immunity level, the administration of the FI-RSV vaccine elicits a CD4 T cell response skewed toward a Th2-type profile. This is characterized by reduced interferon-γ secretion, elevated IL-4 production, IL-13-mediated eosinophil chemotaxis, and IL-4 and IL-10 co-driven pulmonary eosinophilic inflammation, all of which contribute to enhanced immunopathology [16,17,18,19].

Another factor contributing to FI-RSV–induced ERD is the inability of formalin inactivation to preserve the native conformation of the viral F protein. As a result, this antigen will exist in an unstable post-fusion conformation (PostF) [20]. Since PostF does not present key neutralizing epitopes, the vaccine predominantly induces antibodies that can bind to the virus but are ineffective at neutralization. This failure to elicit potent neutralizing antibodies, rooted in the spatial conformational change of the antigen, may represent the key structural basis for the vaccine’s inadequate protection [16,17,20].

## 3. Structure and Function of F and G Proteins

This section delineates the key structural and immunogenic features of the primary RSV antigen targets, the F and G proteins, which form the foundational rationale for the design of the vaccine platforms detailed in the subsequent subsections.

### 3.1. G Protein

**Figure 2:**
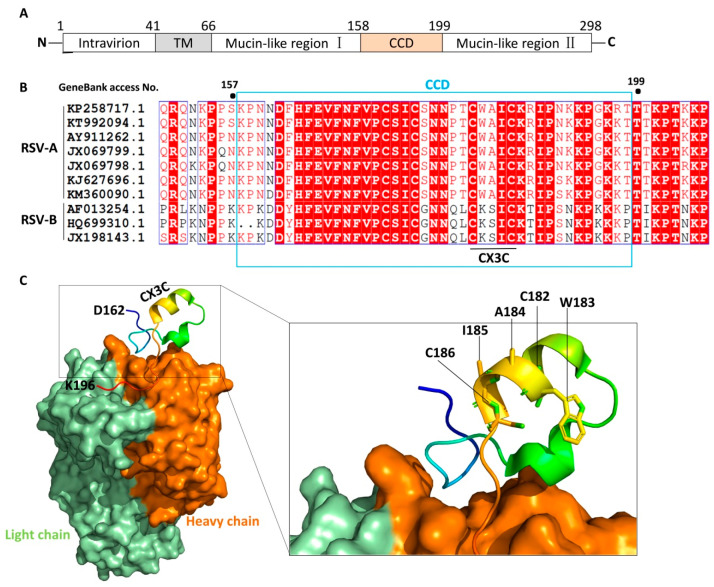
Schematic diagram of RSV G protein structure. (**A**). Schematic representation of the RSV G protein, with its N-terminus located intracellularly and C-terminus extracellularly, anchored to the viral envelope via the TM domain. CCD is located at amino acids 158–199, containing a CX_3_C motif (Cys182–Cys186). Mucin-like regions flanking the CCD are highly variable and glycosylated. (**B**). Sequence alignment of G protein CCD among the indicated strains of RSV. Protein sequences were retrieved from NCBI nucleotide database using the indicated GenBank access numbers. Fully conserved residues have a red background while predominant residues are colored red. (**C**). Schematic diagram of the binding mode between the CCD domain and the neutralizing antibody fragment, Fab CB017.5 (PDB code: 6BLH). The diagram was generated by PyMOL 2.5. The CCD is shown in cartoon representation with the heavy and light chains of antibody colored orange and green, respectively.

The G protein has an alternative start codon in the transmembrane domain, thus it exists in membrane-anchored and secreted forms [21]. The membrane-anchored G protein of RSV mediates immune evasion through three principal mechanisms: it masks key epitopes (e.g., membrane-proximal regions) via transmembrane anchoring to hinder recognition by antibodies or T cells; it subverts immune cell function by inducing immunosuppressive cytokines (e.g., IL-10) through host receptor interactions; and it accumulates surface mutations under immune pressure to facilitate antigenic escape. For vaccine development, this protein poses challenges: steric hindrance and conformational complexity obscure critical neutralizing epitopes, preserving its native conformation when integrated into vectors (e.g., VLPs) is difficult (reducing immunogenicity), and it tends to induce imbalanced responses (e.g., suboptimal antibodies or weak cellular immunity). While some strategies aim to target the secreted G protein (sG)—which can systemically disrupt host immunity through mechanisms like competitive receptor binding—this approach is complicated by the intricate intracellular pathways governing its secretion. Consequently, current research is increasingly focused on targeting its conserved structural domains to counteract immune evasion while minimizing potential off-target effects. Full-length G protein comprises five regions from the N- to C- terminus: the intravirion region, transmembrane (TM) domain, mucin-like region I, CCD, and mucin-like region II (Figure 2A) [22]. The sequence of G protein is highly variable among subgenus of RSV except the CCD domain (Figure 2B). The residues of CCD are partially overlapping with a cystine noose possessing a 1—4, 2—3 disulfide topology, and the residues that span the third and fourth cysteines (Cys182-Cys186) constitute a CX3C motif [11]. It is thought that the CX3C motif mediates attachment of RSV to human epithelial cells by interacting with CX3C receptor 1 (CX3CR1) [11,22]. Structural studies show that the CCD domain maintains a stable conformation with a neutralizing antibody binding site, especially the CX3C motif (Figure 2C), indicating that G protein is a potential target for vaccine exploitation [11].

### 3.2. F Protein

**Figure 3:**
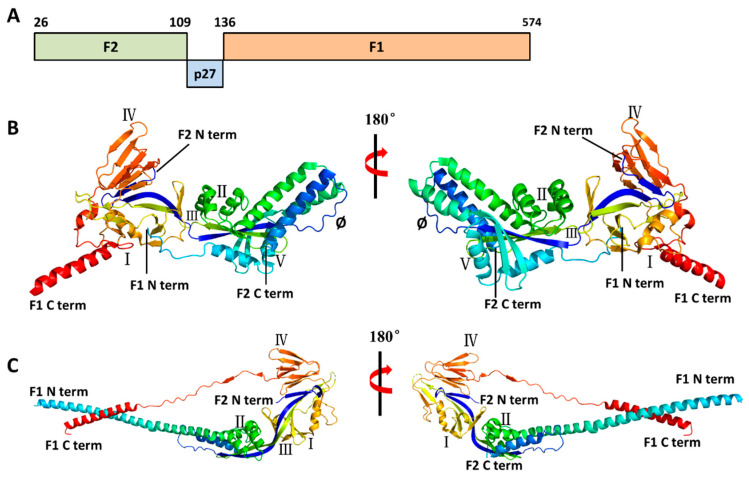
Structures of the RSV F protein in prefusion and postfusion conformations. (**A**). Schematic diagram of RSV F1 and F2 sequences. Cleavage of the F0 precursor protein at residues 109 and 136 generates F2, p27, and F1. (**B**,**C**). Three-dimensional structure of prefusion (PDB code: 4JHW) and postfusion (PDB code: 6APB) F protein. The diagrams were generated by PyMOL 2.5. The six major antigenic sites in prefusion F are labeled as I, II, III, IV, V, and Ø. Compared to prefusion F protein, postfusion protein lacks antigenic site V and Ø.

The F protein is derived from the F0 precursor protein. The F0 protein consists of 574 amino acids, has a polybasic sequence (KKRKRR136), and lacks biological activity [23]. Furin-like proteases can cleave at two polybasic cleavage sites (RARR109 and KKRKRR136) on the F0 precursor protein, generating two subunits, F1 and F2, and simultaneously releasing an internal 27-amino-acid peptide segment called p27 (residues 110–136) (Figure 3A) [24,25]. F protein, which mediates the fusion of the virus with the host cell membrane, has two main conformational states: PreF and PostF. The F1 and F2 proteins can be covalently linked by disulfide bonds to form a heterodimer NH_2_-F2–F1–COOH (Figure 3B), and form a trimer in the endoplasmic reticulum, that is, the metastable PreF conformation [8]. Triggered by unknown factors or thermodynamic instability, the tertiary structure of the PreF protein undergoes extensive rearrangement (Figure 3C). The N-terminus of the protein extends and inserts the hydrophobic fusion peptide of the F1 subunit into the host cell membrane. Subsequently, PreF collapses, and the fusion peptide anchors into the target cell membrane, connecting the virus and the host membrane to form a rod-shaped and stable PostF [26].

Antibodies targeting the F are directed to several specified antigenic sites. Antigenic sites I–IV are shared between PreF and PostF (Figure 3B,C), while epitope Ø and V, which can induce stronger neutralizing antibody activity, are only presented in the PreF conformation, especially site Ø [18,20,26]. PreF has six major antigenic sites (Ø, I, II, III, IV, and V), and PostF has four (I, II, III, and IV). Although approximately 50% of the surface is shared between PreF and PostF, the former is highly antigenic but relatively unstable, while the latter is more stable but less antigenic. The antigenic sites most sensitive to neutralizing antibodies are only located in the prefusion conformation [27].

Although the G and F proteins of RSV are highly glycosylated and may mimic host proteins, they remain prominent antigen candidates in vaccine development for several reasons. A primary reason is that glycosylation does not affect the exposure of key neutralizing epitopes. While glycosylation of G and F proteins may mask some antigenic epitopes, key neutralizing epitopes remain exposed and recognizable by the immune system. For the F protein, highly conserved antigen sites like Ø, III, and V in its PreF conformation are essential for neutralizing antibodies [28], and glycosylation sites (e.g., N27, N70 at F2, and N500 at F1) do not interfere with the conformation of these key epitopes [20]. For the G protein, although its variable regions (N-terminus and C-terminus) are highly glycosylated, the CCD still retains a key CX3C chemokine-mimicking epitope that induces neutralizing antibodies [11]. Furthermore, glycosylation can be optimized to improve immunogenicity. Unnecessary glycosylation sites can be removed (e.g., mutating N-linked glycosylation sites of the F protein such as N27Q, N70Q to reduce glycan masking and improve antibody recognition [28]), while functional glycosylation (e.g., N500 in the F protein that stabilizes protein conformation) needs to be retained to maintain proper antigen folding [20]. Another supporting evidence from natural infection and monoclonal antibody therapy have shown that glycosylated proteins can still induce protective immunity. Humans produce neutralizing antibodies against G and F proteins after RSV infection [29]. Moreover, monoclonal antibodies like Palivizumab (targeting antigen site II of the F protein) and Nirsevimab (targeting antigen site Ø of pre-F) effectively neutralize the virus despite partial glycosylation in the targeted regions [12,24,30]. Finally, advanced vaccine design offers avenues to circumvent potential immune evasion related to glycosylation. Strategies include targeting conserved conformational epitopes (e.g., the Ø site in the pre-F conformation of the F protein which remains exposed despite glycosylation [28]) and leveraging modern platform technologies. For instance, recombinant protein vaccines regulate glycosylation patterns via optimized expression systems [9,10], while mRNA vaccines (e.g., Moderna’s mRNA-1345) enable host cells to self-synthesize antigens, ensuring proper folding and reducing abnormal glycosylation impact [31,32].

Since PreF plays an extremely important role in the immune process, stabilizing the metastable RSV PreF has become a major technical challenge in vaccine development. One example is the development of vaccines that retain the antigenic site Ø. Current methods for stabilizing the structure of RSV PreF include the following approaches. One approach involves introducing cysteine residues at positions 155 and 290 of the RSV F protein (S155C and S290C), enabling the formation of a stabilizing disulfide bond. This disulfide-stabilized (DS) mutant prevents the protein from transitioning to the post-fusion state while retaining antigenic site Ø and promoting stable trimer formation. Another strategy is cavity filling, where hydrophobic residues are inserted to occupy internal cavities of the protein, thereby enhancing van der Waals interactions and overall structural rigidity. For instance, Cavity-filling mutation 1 (Cav1) S190F and V207L fill specific hydrophobic cavities in the RSV F protein to form stable RSV F trimers and retain the antigenic site Ø. Similarly, the F488W mutation and triple-cavity-filling (TriC) mutation set D486H-E487Q-F488W-D489H stabilize the region near the fusion peptide with a slightly lower expression level than that of Cav1. Extra mutations, including K87F-V90L, S190F-V296F, and V207L-V220L, can enhance the recognition by antibody D25, yet their expression levels remain low. To further improve stability, combinatorial optimization approaches have been used. The combination of DS and Cav1 mutations generates the DS-Cav1 variant, which shows superior performance in terms of trimer yield and physical stability against temperature fluctuations, pH changes, osmotic pressure, and freeze-thaw cycles. Combining DS or Cav1 with the TriC mutation (DS-TriC or Cav1-TriC) confers further stability, while the DS-Cav1-TriC combination, combining all three mutations (S155C-S290C, S190F-V207L, D486H-E487Q-F488W-D489H), forms the most stable variant. Other approaches include the addition of N-linked glycosylation sites—such as introducing a V178N mutation—to improve structural stability. However, this method fails to stabilize the antigenic site Ø. Likewise, targeted postfusion destabilization mutations (e.g., V185E and I506K) disrupt the stability of the postfusion state, thereby enhancing the stability of the prefusion state, but these mutations fail to stabilize the antigenic site Ø. Finally, trimerization of the RSV F protein can be enhanced by fusing the T4 bacteriophage fiber protein (“foldon”) to the C-terminus of the extracellular domain of RSV F [30]. The foldon domain serves as a trimerization motif that ensures the antigen forms a stable trimer. However, it should be noted that the foldon domain alone cannot stabilize the prefusion conformation; rather, it provides a necessary foundation for subsequent stabilization by specific amino acid mutations.

## 4. RSV Vaccine Strategies: Subunit and VLP Approaches

This section outlines two predominant strategies for RSV vaccine development: subunit vaccines and particle-based vaccines, particularly virus-like particles. For each approach, we summarize the design principles, clinical development progress, and core advantages and disadvantages.

### 4.1. Subunit Vaccines

Subunit vaccines are formulated using specific proteins (subunits) of the virus instead of the complete viral entity and are devoid of viral genetic material. Such vaccines typically incorporate the F protein of RSV or other immunogenic proteins, which can stimulate the body to produce an immune response. The advantage of subunit vaccines lies in their favorable safety profile, as the absence of live viruses eliminates the risk of vaccine-induced infection. This characteristic makes them suitable for prophylactic immunization in target populations such as pregnant women and the elderly.

#### 4.1.1. Abrysvo (RSVPreF)

Abrysvo, developed by Pfizer, is a bivalent subunit vaccine based on the PreF, which exists in a stable form and provides active immunization against two subtypes, RSV-A and RSV-B [31]. Approved for marketing by the US FDA in 2023, Abrysvo is indicated for the prevention of LRTI caused by RSV in pregnant women and people aged 60 and above. Each dose of the vaccine contains 120 μg of the prefusion-stabilized RSV F protein, with 60 μg of the PreF protein for each of the RSV-A and RSV-B subtypes. These antigens are expressed in genetically engineered Chinese hamster ovary cells and formulated into the vaccine after purification [33]. The prefusion-stabilized RSV F antigen developed by Pfizer consists of the RSV F ectodomain structurally stabilized by a C-terminal T4 phage fibritin foldon domain to enforce trimerization, combined with internal stabilizing mutations to lock the conformation in the prefusion state [34]. The development of the Abrysvo has gone through multiple phases of clinical trials. Since newborn babies and the elderly are more vulnerable to RSV, the impact of the vaccine on these groups has been studied in depth in various trials [31].

Numerous clinical trials of Abrysvo have confirmed its high safety and efficacy in pregnant women and the elderly. The Phase III, double-blind MATISSE study (Maternal Immunization Study for Safety and Efficacy) trial evaluated maternal immunization with the RSV vaccine to prevent RSV-associated LRTI in neonates and infants. Its results demonstrated that within 90 days after birth, the incidence of severe RSV-associated LRTI requiring medical attention was significantly lower in the vaccine group than in the placebo group, showing a vaccine efficacy of 81.8%. Within 180 days, the efficacy remained significant at 69.4% [35]. The results of the GRADE (Grading of Recommendations, Assessment, Development and Evaluation) assessment of Abrysvo submitted to the Advisory Committee on Immunization Practices (ACIP), showed that in all trial populations (with a trial dosing interval of 24–36 weeks of pregnancy), the efficacy against RSV-related LRTI was 51.3%, while the efficacy of pre-vaccinating the mother with the RSV vaccine during the approved dosing interval (32–36 weeks of pregnancy) was 57.3% [32,36]. In addition, Abrysvo has shown favorable efficacy in the elderly population. The ConquerRSV Study conducted by the ACIP of the Centers for Disease Control and Prevention (CDC) in the United States mentioned that in four observational studies, the protective efficacy of this vaccine against RSV-related hospitalizations was 75% to 82% in adults aged 60 and above, and it also showed effectiveness in immunosuppressed adults and patients with end-stage renal disease [32]. Other Phase I/II randomized clinical studies have further established the safety and immunogenicity of Abrysvo, confirming its ability to induce a strong RSV-neutralizing antibody response [37].

In summary, Abrysvo demonstrates outstanding performance in preventing RSV, especially in pregnant women and the elderly population. For pregnant women, the advantages of the Abrysvo include providing early and immediate protection to infants after birth through maternal immunization, and significantly reducing the risk of RSV-related LRTI hospitalization [31,32]. While there are potential risks of preterm birth and hypertensive disorders of pregnancy, ACIP deems these risks acceptable when the vaccine is given between 32 and 36 weeks of gestation [38]. For the elderly population, given the vaccine’s favorable safety and efficacy profile, ACIP recommends that all individuals aged 75 years and older, as well as those aged 60–74 who are at increased risk of severe RSV disease, receive this vaccination [32].

#### 4.1.2. Arexvy (RSVPreF3)

Arexvy (RSVPreF3) is an RSV vaccine developed by GlaxoSmithKline (GSK). The US FDA approved the marketing of Arexvy (RSV adjuvanted vaccine) on 23 May 2023. This vaccine has shown significant potential in preventing RSV-related LRTI in individuals aged 60 and above [39,40,41,42]. Arexvy utilizes recombinant subunit technology to stabilize the RSV F protein in the PreF conformation, in combination with the AS01E adjuvant to enhance immunogenicity [41,42]. RSVPreF3, the main component in Arexvy, is a recombinant F protein. Chinese hamster ovary cells are genetically engineered to express PreF in a stable conformation, aiming to induce or enhance the RSV PreF-specific antigen site (Ø and V)-binding antibody response [43]. The AS01E adjuvant is composed of 3-O-heptanoyl-4′-monophosphoryl lipid A (MPL) derived from *Salmonella minnesota* and QS-21—a saponin isolated and purified from the plant *Quillaja Saponaria Molina*. These two components are mixed in liposomes made up of di-oleoylphosphatidylcholine (DOPC) and cholesterol [44,45]. This adjuvant system boosts the immune response and enhances vaccine efficacy, optimizing vaccine performance.

The AS01E adjuvant offers multiple advantages. AS01E, which contains MPL and QS-21, induces a broader neutralizing antibody response with increased breadth and titer. It can activate the innate immune system, such as the TLR4 signaling pathway, to promote polyclonal B cell activation and antibody affinity maturation. This leads to the production of highly potent neutralizing antibodies against multiple RSV strains, including RSV-A and RSV-B subtypes [45]. In contrast, the alum adjuvant mainly enhances Th2 humoral immunity, but has a narrow range of antibody response and a weak neutralizing effect on clinical isolates of the RSV-B subtype [43]. For enhancing the multifunctional CD4^+^ T cell response, including Th1 bias and cellular immunity, AS01E synergistically activates dendritic cells through MPL (a TLR4 agonist) and QS-21 (a saponin). This activation promotes the secretion of IL-12 and induces the differentiation and versatility of Th1 CD4^+^ T cells (e.g., simultaneous secretion of IFN-γ, IL-2, and TNF-α) [45]. However, the alum adjuvant mainly induces Th2-type responses (such as the production of IL-4 and IL-5) and has a weak effect on enhancing the multifunctionality and Th1 bias of CD4^+^ T cells [17,46]. To overcome immunosenescence in the elderly, AS01E promotes the germinal center response by activating innate immunity, reverses age-related decline in T cell function, and reactivates memory B cells and follicular helper T cells (Tfh) [46]. In contrast, alum adjuvants have a weak effect on cellular immunity in the elderly, making it difficult to effectively overcome immune aging [41,46]. To enhance antigen presentation through liposomal delivery systems, AS01E encapsulates MPL and QS-21 within liposomes (including DOPC and cholesterol), which promotes the uptake of antigens by antigen-presenting cells (APCs) and enhances cross-presentation [45]. In contrast, the alum adjuvant releases antigens from a deposition site but cannot effectively activate cellular immunity or promote cross-presentation [45].

As a vaccine that has been launched on the market, Arexvy has undergone rigorous evaluation in multiple clinical trials. The FDA recommends Arexvy for individuals aged 60 and above, and for those aged 50 to 59 with an increased risk of RSV-related LRTI [44]. In a placebo-controlled Phase III clinical study (NCT04888596, name: AReSVi-006), adults aged 60 and above participated and received either a single dose of Arexvy (containing RSVPreF3 antigen and AS01E adjuvant) or a placebo. The efficacy of the vaccine against RT-PCR-confirmed LRTI caused by RSV was 82.6% (96.95% confidence interval [CI], 57.9% to 94.1%), and the efficacy against severe RSV-related LRTI was 94.1% (95% confidence interval, 62.4% to 99.9%) [41,44,45]. Common adverse reactions include pain at the injection site, fatigue, and headache, but most of them are mild or moderate. The incidence of serious adverse events occurring within 6 months after vaccination was similar in participants who received AREXVY (4.2%) or placebo (4.0%) [41,44]. Further research showed that the efficacy of a single-dose vaccine in two RSV seasons was 67.2% (97.5% confidence interval of 48.2% to 80.0%). The efficacy of the vaccine against RSV-related LRTI in two seasons when vaccinated again 1 year after the first vaccination was 67.1% (97.5% confidence interval of 48.1% to 80.0%), and the efficacy against severe LRTI was 78.8%. These results indicate that a single-dose Arexvy vaccine has acceptable safety in individuals aged 60 and above and is effective against RSV-LRTI in at least two RSV seasons [42]. In addition, the results of a Phase III, observer-blinded, randomized, placebo-controlled study (NCT05590403) with participants aged 50 to 59 (*n* = 769 for Arexvy; *n* = 383 for normal saline placebo) showed that within 6 months after vaccination, 2.3% of individuals who received Arexvy reported serious adverse events, compared with 2.1% in the placebo group. Moreover, comparative analysis of neutralizing antibody responses to RSV-A and RSV-B demonstrated that adults aged 50 to 59 with chronic diseases achieved neutralizing antibody titers and seroresponse rates comparable to those observed in individuals aged 60 and above, thereby meeting the immunobridging criteria and supporting an effective immune response in this age group [44]. In conclusion, Arexvy shows good safety and can induce an effective immune response in individuals aged 60 and above, as well as in those aged 50 to 59 with an increased risk of RSV-related LRTI, reducing the risk of diseases caused by RSV infection.

The main advantages of Arexvy include high efficacy, a broader indicated population compared to some alternatives, and the use of the AS01E adjuvant which enhances the immune response to provide safer and more effective protection for the elderly population [39,40,44,45]. However, a potential risk associated with Arexvy is GBS. An analysis from the FDA’s self-controlled case series estimated the risk of GBS in Medicare beneficiaries aged 65 and above due to vaccination with the RSV subunit vaccine. The adjusted incidence rate ratio of GBS for Arexvy in the risk interval compared to the control interval was 2.30 (95% CI, 0.39 to 13.72), while for Abrysvo it was 4.48 (95% CI, 0.88 to 22.90) [32]. In addition, Arexvy needs to be stored in a refrigerator at 2 °C to 8 °C before use and can only be stored at room temperature for 4 h, which may limit the promotion and use of this vaccine in some areas [44]. Nevertheless, Arexvy still has good safety and high efficacy, and can provide significant protection for the elderly population.

#### 4.1.3. DS-Cav1

DS-Cav1 was jointly developed by the National Institutes of Health (NIH) and National Institute of Allergy and Infectious Diseases (NIAID) in the United States [28]. Analysis of human serum samples demonstrates that most neutralizing antibodies elicited by natural RSV infection bind to PreF as their primary target. PreF has six antigenic sites (I, II, III, IV, V, Ø), among which sites V and Ø located at the distal vertex of the cell membrane are the target sites of many potent antibodies. To enhance the induction of similar effective antibodies, researchers designed RSV F soluble variants, including DS-Cav1, that can stably expose antigenic site Ø as RSV vaccines [47].

The Ø site of PreF can be stabilized by attaching the foldon region to the C-terminus of the extracellular domain of RSV F [48,49] and binding to the D25 antibody. The D25 antibody is a high-affinity human monoclonal antibody targeting RSV, generated by retrovirally transducing *BCL6* and *Bcl*-*xL* into human peripheral blood CD27^+^ memory B cells and screening via RSV microneutralization assays. D25 is important to combat RSV infections in high-risk populations such as infants and the elderly. It demonstrates superior in vitro potency (IC_50_ 2.1 ng/mL against RSV-A2, ~100-fold more effective than palivizumab) and broad activity against clinical isolates like RSV-X. In vivo, it offers strong prophylactic efficacy in cotton rats, achieving full lung viral inhibition at 0.6 mg/kg (versus 2.0 mg/kg for palivizumab) [50]. However, the D25 antibody blocks the Ø site. To stably present the Ø site without the D25 antibody, researchers introduced DS and Cav1 to PreF. DS refers to the introduction of the S155C-S290C double mutation, where the cysteine residues at positions 155 and 290 form a disulfide bond, preventing the conversion of PreF to PostF. However, the membrane-distal part (including the Ø site) in the DS variant remains relatively disordered or adopts multiple conformations, and this variant also exhibits the lowest stability in response to pH and temperature changes [28]. Cav1 refers to the introduction of the S190F-V207L mutation, where the hydrophobic groups fill the cavity in the membrane-distal head of PreF, and the structure at the membrane-distal vertex is more ordered [28]. When it comes to yield and physical resistance to extreme temperatures, pH values, osmotic pressure, and freeze-thaw cycles, the DS-Cav1 combination is found to be optimal. Furthermore, negative stain electron microscopy confirmed that the DS-Cav1 proteins form a homogeneous population [28].

Researchers from the Vaccine Research Center, NIAID, NIH conducted a Phase I, open-label, randomized, single-center domestic clinical trial. The aim was to evaluate the dosage, safety, tolerability, and immunogenicity of the investigational prefusion-stabilized RSV F subunit protein vaccine DS-Cav1 [51]. Ninety-five subjects received the DS-Cav1 vaccine, administered with or without alum adjuvant, at weeks 0 and 12. The dosage of the two vaccinations was the same, and they were followed up until week 44. The DS-Cav1 subunit vaccine was safe and well-tolerated at doses up to 500 μg. After the completion of the trial at week 44, a single dose of the vaccine was proven to have persistent virus-neutralizing activity, indicating that DS-Cav1 may play a protective role in more than one RSV epidemic season. Notably, the mother-to-infant transfer ratio of RSV-specific IgG antibodies induced by DS-Cav1 is close to 1:1, and a single vaccination in the second or third trimester of pregnancy may result in the newborn’s neutralizing antibody activity being more than 10 times the general level—this could extend infant protection to the first six months after birth, when airway development and immune maturation reduce RSV infection severity [52,53,54,55,56,57]. However, concerns remain that maternally derived hyperactive antibodies might affect infant lung development and function, though this has not been confirmed in clinical trials [49].

Despite its advantages, DS-Cav1 exhibits significant limitations in structural stability and immunogenicity, especially in the context of aging and cold-chain dependence. After 5 weeks of storage at 4 °C, DS-Cav1 loses 82% of its binding affinity for the PreF-specific antibody AM14 (which targets the V epitope), and its induced neutralizing antibody titer in mice is only 1/11 that of DT-PreF [58]. In addressing immunosenescence in the elderly—a key high-risk group for RSV—preclinical studies in aged mice (17 months old) show that DS-Cav1 induces both humoral and cellular immune responses that are significantly inferior to those in young mice. The number of RSV F-specific IFN-γ-secreting CD8^+^ T cells is reduced by 50%, and the expression of B cell activation markers (e.g., CD86) is decreased by 40% [59]. Even when combined with the TLR4 agonist adjuvant GLA-SE (glucopyranosyl lipid A formulated in a stable emulsion), the cellular immunity level of aged mice is only slightly enhanced, remaining far lower than that induced in young mice—indicating that DS-Cav1 alone is insufficient to overcome age-related immune decline [59]. This immunosenescence-related defect may explain why DS-Cav1 has not yet advanced to late-stage clinical trials in the elderly population.

In summary, DS-Cav1 is a stable mutant of PreF. As a PreF subunit vaccine, its main target groups are pregnant women and the elderly. Although it showed good tolerance and immunogenicity in healthy adults in Phase I clinical trials and has the potential to protect newborns, the highly potent antibodies produced by the mother may cause sequelae in the development and function of the infant’s lungs [49]. In addition, research on the efficacy of the vaccine for the elderly is currently lacking. Preclinical studies in aged mice have revealed that the humoral and cellular immunity induced by DS-Cav1 is substantially lower than that in young mice. The adjuvant GLA-SE (glucopyranosyl lipid A formulated in a stable emulsion, a TLR4 agonist) only slightly enhanced the cellular immunity in aged mice, and the response remained far weaker than that induced in young mice. Therefore, the old mouse model may be a useful tool for evaluating and improving the vaccine, aiming to prevent RSV infection in the elderly [59].

#### 4.1.4. DT-PreF

DT-PreF is an RSV subunit vaccine developed by Calder Biosciences. It is a stable mutant of RSV PreF, distinguished by its unique structural modification—dityrosine bonds (DT bonds), which are zero-length covalent bonds formed by radical-mediated oxidative cross-linking of tyrosine (Tyr) side chains. Unlike conventional disulfide bonds, dityrosine bonds exhibit superior chemical stability. They can withstand extreme pH conditions (pH 2–10) and boiling in reducing buffers at 100 °C without degradation. Their formation relies on an enzyme-catalyzed system: Arthromyces ramosus peroxidase (ARP) triggers Tyr cross-linking in the presence of hydrogen peroxide (H_2_O_2_) via a resonance-stabilized radical mechanism, and this reaction can be real-time monitored using its intrinsic fluorescence (excitation wavelength: 320 nm, emission wavelength: 400 nm) [60,61]. This “post-folding modification” mechanism—occurring only after the protein adopts its native conformation—avoids misfolding and abnormal aggregation common in disulfide bond-engineered proteins (e.g., DS-Cav1) [58].

DT-PreF contains two dityrosine bonds, which stabilize the molecule in the PreF conformation to elicit neutralizing antibody responses against specific epitopes (Ø and the IV/V interface). The two engineered dityrosine bonds are precisely targeted to key structural domains of PreF. One is formed by the pairing of V185Y and N428Y, which stabilizes the IV/V interface. The other is formed by K226Y and the endogenous tyrosine Y198, which locks the core neutralizing epitope Site Ø. These modifications ensure that critical neutralizing epitopes remain exposed and prevent PreF-to-PostF conformational transition [58]. Additionally, DT-PreF includes three point mutations (L512V, L513V, Y519F) located in the foldon region, which further enhance trimer stability without interfering with epitope accessibility [58].

The stability of DT-PreF under low-temperature conditions is significantly higher than that of DS-Cav1. Differential scanning fluorimetry analysis shows that the melting temperature (Tm) of DT-PreF is 78 °C, which is 25 °C higher than that of uncross-linked PreF and 17 °C higher than that of DS-Cav1 [3]. Immunological experiments (ELISA) and animal studies confirm that DT-PreF maintains a stable prefusion conformation and intact immunogenicity after incubation at 4 °C for 11 weeks (verified by 5C4/AM14 double-antibody sandwich ELISA). In contrast, DS-Cav1 underwent significant conformational changes after just 5 weeks at 4 °C, resulting in an 82% loss of AM14 binding capacity and a 57.6% reduction in the neutralizing antibody titers it induced [3,58]. Even under short-term ambient temperature storage (25 °C for 7 days), DT-PreF retains 85% of its prefusion conformation, whereas DS-Cav1 only retains 42% and can no longer induce an effective neutralizing response [58]. This high stability directly reduces dependence on cold-chain logistics—a critical advantage for low-and middle-income countries with limited cold-chain infrastructure: in simulated transportation experiments (exposure to 30 °C for 4 h + 1000 rpm vibration for 2 h), DT-PreF maintains 90% prefusion purity, while DS-Cav1’s purity drops to 58% with a 60% loss of neutralizing activity. Furthermore, after opening, DT-PreF remains sterile and immunogenic for 72 h at 4 °C, extending the in-use shelf life compared to conventional vaccines (typically 24 h) and reducing vaccine waste in remote areas [58].

DT-PreF has not yet entered clinical trials, but animal studies show that DT-PreF exhibits a powerful protective efficacy in cotton rats. Administration of 2 μg and 10 μg of DT-PreF renders RSV undetectable in the lung tissues of cotton rats, and induces the immune responses against RSV A and RSV B that are both stronger than those produced by natural infection. Notably, DT-PreF addresses a key challenge of RSV vaccines in the elderly—overcoming immunosenescence—by simply increasing the antigen dose: in 17-month-old aged mice, a 4.5-fold dose of DT-PreF (45 μg) elicits a neutralizing antibody titer (geometric mean titer [GMT] = 19,800) that is not statistically different from that induced by a 10 μg dose in 4-month-old young mice (GMT = 21,206, *p* > 0.05). This effect is achieved using only alum adjuvant, without the need for poorly tolerated high-potency adjuvants or repeated administration—avoiding adverse reactions associated with adjuvants in the elderly [58]. In terms of immunogenicity, DT-PreF induces a neutralizing antibody titer 11 times higher than that of DS-Cav1 in mice, and in cotton rats, it achieves “sterile lung protection” (undetectable viral load after wild-type RSV challenge) against both RSV-A (Tracy strain) and RSV-B (18537 strain)—a broad-spectrum effect attributed to its stabilization of conserved epitopes (Site Ø and IV/V, sequence conservation > 90%) [58].

While DT-PreF has not been evaluated in maternal immunization studies, its stable PreF conformation and high antibody-inducing capacity suggest potential for transplacental protection: if maternal vaccination induces high-titer neutralizing antibodies, the FcRn-mediated transfer efficiency (similar to Abrysvo’s 1.46-fold umbilical cord-to-maternal titer ratio) could provide infants with passive protection for 6 months. However, this requires validation in clinical trials [61,62].

In summary, DT-PreF has stronger low-temperature stability compared to other RSV subunit vaccines, which extends its shelf life and makes it easier to transport. DT-PreF is more effective, inducing a neutralizing antibody titer 11 times higher than that of DS-Cav1. It can overcome immunosenescence in a mouse model merely by increasing the dose, suggesting that the vaccine may more easily achieve protective effects in the elderly population. DT-PreF is also highly effective in cotton rats, inducing high neutralizing antibody titers against RSV strains A and B and achieving lung sterility upon wild-type RSV challenge [58]. However, due to the current lack of clinical studies on DT-PreF, whether and when it can be marketed remains unknown.

#### 4.1.5. ADV110

ADV110 (BARS13) is a recombinant protein subunit vaccine for RSV developed by Suzhou Advaccine Biotechnology Co., Ltd. (Suzhou, China). It uses the novel adjuvant AE011 and aims to prevent RSV-induced respiratory-related diseases. Different from most of the vaccines under development targeting the F protein, the ADV110 targets the G protein of RSV. The G protein has relatively stable neutralizing epitopes and is relatively independent of its protein structure. The function of the G protein is to act as an attachment protein during the RSV infection process. It promotes the adsorption of the virus by interacting with the receptors of target cells [63].

The RSV envelope G protein contains a central CCD of approximately 40 amino acids (amino acids 162–196). This domain lacks glycosylation and plays a crucial role in viral infection and pathogenicity. The CCD contains a CX3C motif (amino acids 182–186), which helps bind to the CX3CR1 receptor on cells, leading to RSV infection in human respiratory epithelial cells. Previous studies have shown that the CCD domain of RSV G protein is an exposed region that can bind to antibodies. Antibodies against this region can exhibit strain-independence and treat RSV infection in human respiratory airway epithelial cells [64]. ADV110 was designed based on this CCD domain. In the design of the ADV110 vaccine, cyclosporin A (CsA) is added. By inducing specific neutralizing antibodies against the RSV G protein and antigen-specific regulatory T cells (Tregs), it prevents the virus from entering cells and inhibits RSV infection [63]. At the same time, the adjuvant AE011 can regulate the immune response and reduce excessive T-cell responses. Immunization of BALB/c mice shows that the antibody response is dose-dependent, and a detectable antibody response can still be induced at the lowest dose (30 ng). The viral load in the lungs of all mice immunized with the G-protein vaccine is significantly lower than that of the non-immunized group, verifying the efficacy of the ADV110 vaccine [22].

In the Phase II clinical trial announced by Advaccine, both the low-dose recipients (LDR) and high-dose recipients (HDR) exhibited significantly higher median IgG antibody levels compared with the placebo recipients. On day 30 post-vaccination, the median antibody concentrations of LDR and HDR were 763.18 IU/mL and 885.74 IU/mL, respectively. By day 60, the antibody concentrations of LDR and HDR were both higher than the baseline, with median values of 1187.10 IU/mL and 1482.12 IU/mL, respectively. After receiving 1 or 2 doses of ADV110, the antibody concentrations of all vaccine recipients showed a clear upward trend. Especially for the participants using the two-dose regimens (LDR and HDR), the increase in their IgG antibody concentrations on day 60 was much higher than that on day 30. In addition, flow cytometry analysis in human clinical trials found that after vaccination with ADV110, the PBMCs stimulated by a specific peptide of G protein (G-peptide) did not significantly activate Th1/Th2 cytokines (such as IFN-γ, TNF-α), while the positive control group stimulated by CD3/CD28 showed an obvious response. This indicates that the Treg induction mechanism of ADV110 may inhibit some antiviral T-cell responses [63]. ADV110-induced Treg activation poses dual effects on antiviral immunity: It may suppress effector T cells (e.g., Th1, CTL), reducing IFN-γ and viral clearance; yet moderate activation could limit excessive RSV-induced lung inflammation, balancing protection and pathology. Furthermore, long-term implications depend on Treg activity levels. Moderate Treg activation may sustain immune homeostasis, particularly in the elderly. However, Treg overactivation risks prolonged viral replication, heightened reinfection susceptibility, blunted responses to other vaccines, and impaired immune memory—weakening long-term protection against RSV reinfection.

Regarding safety, the most common systemic adverse reactions among recipients of each dose were fatigue, headache, myalgia, and discomfort. The incidence of fatigue, headache, and discomfort among placebo recipients was relatively high, with 5, 7, and 3 cases reported, respectively [65]. In terms of cellular immunity, flow cytometry analysis revealed that peripheral blood mononuclear cells (PBMCs) from vaccinated individuals did not mount a significant response upon stimulation with the G protein peptide. In contrast, a robust response was observed in samples stimulated with CD3/CD28. This indicates that the ADV110 vaccine does not provoke excessive T-cell activation, thereby potentially lowering the risk of vaccine-enhanced disease (VED) [63].

Currently, the ADV110 has completed Phase I and Phase II clinical trials. The Phase I study focused primarily on assessing the safety, tolerability, and initial immunogenicity. The Phase II trial further evaluated the safety and immunogenicity of the vaccine in a larger population and explored the effects of different doses and vaccination regimens. The Phase II clinical trial showed that the ADV110 vaccine exhibited good safety and immunogenicity in adults aged 60–80, and significantly increased the antibody level against RSV [66]. Unlike the common F-protein antigen vaccines, the ADV110 is a G-protein–based vaccine that innovatively uses AE011 as an adjuvant. By regulating the immune response, the AE011 adjuvant reduces excessive T-cell responses and the related risks of VED.

### 4.2. Particle-Based Vaccines

VLPs are multi-protein complexes that can self-assemble and have a structural similarity to the viral capsid [67]. According to the presence or absence of an envelope, VLPs can be divided into enveloped and non-enveloped types; according to the number of layers, they can be further divided into single-layer, double-layer, and multi-layer VLPs [68]. VLPs can induce a relatively strong immune response, including cellular and humoral immunity [69]. However, due to the lack of genetic material, VLPs cannot replicate independently or infect cells, which makes VLP-based vaccines theoretically safer than traditional vaccines. RSV VLPs are non-replicating nanoparticles approximating dimensions of 80–400 nm. These particles comprise a host-derived lipid bilayer devoid of genomic RNA, functional ribonucleoprotein complexes, or native filamentous architecture. Surface immunogens—predominantly prefusion-stabilized F protein trimers and membrane-anchored G glycoproteins retaining CX3C chemokine motifs—embody key neutralization epitopes while avoiding conformational dynamism inherent to wild-type counterparts.

Structural integrity of RSV vaccine candidates can be achieved via two major strategies. One relies on the scaffold-like oligomerization of RSV M protein, which interacts with glycoprotein cytoplasmic tails to form pleomorphic assemblies. The other employs heterologous platforms (e.g., HIV Gag or NDV glycoprotein chimeras) enforcing geometric spacing and prefusion stability via transmembrane-cytosolic domain substitutions. Cryo-electron tomography confirms spherical topologies bearing surface-exposed glycoprotein spikes (~10–15 nm projections), yet starkly contrasting with authentic virions by lacking nucleocapsid helicity, size heterogeneity, and membrane curvature dynamics. Critically, both platforms leverage optimal particle diameters (80–300 nm) for size-dependent lymphatic trafficking and dendritic cell uptake. Their repetitive antigen display achieves >100-fold higher epitope density than subunit vaccines, driving potent B-cell activation while circumventing conformational instability seen in soluble trimeric antigens. This coordinated design integrates epitope spatial organization with biophysical stability to overcome limitations of conventional vaccine approaches [70].

Compared with other subunit vaccines, VLP vaccines can provide more antigenic epitopes, thus significantly enhancing antibody reactivity and the immune response [71]. These advantages make VLPs a highly promising biological tool, showing great application prospects in the field of vaccine research and development. VLP vaccines and subunit vaccines are often classified into the same category. The main reason is that they are both based on some structural components of the virus rather than the complete pathogen. In addition, both rely on specific viral proteins as their core components. They have no virus replication and no risk of infection, and there is an overlapping part in certain stages of vaccine development.

Comparative analyses [72] indicate that VLP vaccines co-targeting both F and G proteins confer superior neutralization breadth and longevity relative to those exclusively targeting prefusion F protein. Prefusion F-specific vaccines (e.g., mRNA-LNP platforms) elicit high-titer neutralizing antibodies against dominant antigenic sites (sites Ø/V), achieving >94% efficacy against severe disease and sustained GMFR > 3.2 at 12 months in older adults. In contrast, dual-antigen VLPs incorporating RSV F and G glycoproteins demonstrate qualitatively enhanced immunogenicity. Specifically, these VLPs induce 4–10-fold higher cross-neutralizing titers against diverse RSV strains. This synergistic effect stems from complementary epitope presentation: prefusion F provides potent neutralization determinants, whereas G protein contributes conserved CX3C chemokine motifs and Th-cell epitopes that augment mucosal immunity and durable memory B-cell responses. Mechanistically, F+G VLPs mitigate VED risk by promoting balanced Th1 polarization (IFN-γ^+^ CD8^+^ T cells) and reducing IL-4-driven pathology, while extending protective durability beyond 18 months in preclinical models—significantly outperforming F-only subunit vaccines in both potency and longevity across age groups.

IVX-A12 is a bivalent VLP vaccine developed by Icosavax. It is specifically designed for two major pathogens that cause LRTI in adults aged 60 and older, including RSV and human metapneumovirus (hMPV). This vaccine combines two VLP components, IVX-121 and IVX-241, which target RSV and hMPV, respectively. IVX-121 was originally a subunit vaccine developed based on VLP technology, targeting PreF conformation of RSV F protein [73]. It has completed a 12-month immunogenicity Phase I/Ib clinical trial. Data demonstrated that a single dose of IVX-121 induced a durable neutralizing antibody response against RSV that persisted for 12 months. In addition, preliminary evidence from a Phase Ib extension trial indicates that subjects who received 75 μg of adjuvant-free IVX-121 one year after the initial vaccination had a strong immune response to RSV-A [74]. Currently, this vaccine, as part of IVX-A12, is undergoing further clinical trials together with IVX-241, a subunit vaccine targeting the PreF form of hMPV.

The Phase II clinical trial data of the IVX-A12 vaccine indicate that it induces a strong immune response against both RSV and hMPV. RSV-A/B neutralizing antibodies increased 2- to 6-fold by Day 28 with the GMT reaching 12,200 IU/mL compared with 2000 IU/mL in placebo. hMPV-A/B antibodies rose 2.3- to 4-fold with no significant change in placebo groups, attributable to high-density antigen presentation driving potent B-cell activation and cross-protective immunity [75,76]. In addition, IVX-A12 was generally well-tolerated in the trial, and its safety profile was similar to that of the Phase I trial.

As the first RSV/hMPV combination candidate to advance to Phase III trials, it uniquely mitigates healthcare burdens through combined prevention, particularly for high-risk populations. Leveraging VLP technology, IVX-A12 demonstrates high immunogenicity, with Phase II trials confirming robust responses. However, the vaccine faces challenges including complex manufacturing and elevated costs due to the precise assembly requirements of VLP structural proteins, which complicate scalability compared to subunit platforms. While Phase II data showed no significant immune interference between antigens, Phase III trials must conclusively validate balanced immunogenicity to rule out antigen competition risks. Furthermore, long-term protection remains unvalidated as current efficacy data extend only to Day 365 post-vaccination, leaving durability, booster requirements, and real-world effectiveness against evolving strains unconfirmed pending Phase III results.

Overall, IVX-A12 demonstrates high levels of safety and efficacy, and has the potential to become the world’s first dual-target vaccine against RSV and hMPV, providing a new solution for preventing LRTI in young and elderly people.

## 5. Discussion

In recent years, significant progress has been made in the development of RSV subunit vaccines (Table 1). In particular, the PreF has become the primary focus of current research due to its high immunogenicity and full exposure of neutralizing epitopes. Among the vaccines reviewed in this article, Abrysvo and Arexvy, as representative products already on the market, have confirmed the feasibility and clinical value of PreF conformational stability [35,41]. Candidate vaccines such as DS-Cav1, DT-PreF, and IVX-A12 have further optimized stability and immunogenicity through molecular engineering techniques. In addition, the ADV110 vaccine targeting the G protein has achieved initial results in clinical trials [66]. However, there are still multiple challenges to overcome in the process of translation from the laboratory to the clinic.

Historically, the failure of clinical trials of the FI-RSV vaccine revealed the potential harm of non-neutralizing antibodies that cause VED. This lesson has informed current design of subunit vaccines to pay more attention to the precise control of the PreF conformation to avoid eliciting non-functional or potentially harmful antibodies. However, there are other risks in the vaccines currently under development. Post-marketing surveillance of Arexvy and Abrysvo has indicated a slightly increased risk of GBS, with adjusted incidence rate ratios of 2.30 and 4.48, respectively [32]. Although the causal relationship has not been established, the FDA has required the update of product labels to add corresponding statements about GBS. In addition, the G-protein-based vaccine ADV110, which targets the CCD, also presents potential safety concerns. Although it reduces the viral load in the lungs in animal models, its Treg activation mechanism may inhibit antiviral immunity, and its safety needs to be verified through clinical trials.

The metastable property of the PreF protein is a core challenge in vaccine design. Abrysvo “locks” PreF in the prefusion state through the foldon region and internal stabilizing mutations, preserving key antigenic sites (such as Ø and V), thus inducing a high-efficiency immune response [34]. Similarly, DS-Cav1 significantly enhances the thermal stability and conformational transition resistance of PreF through the synergistic effect of DS and Cav1 [30]. DT-PreF, which incorporates di-tyrosine bonds (V185Y/N428Y and K226Y/Y198), exhibits better stability than DS-Cav1 at low temperatures, thereby offering new possibilities for vaccine storage and transportation [58]. Although the above-mentioned strategies show promise in preclinical trials in animal models and early clinical trials, they still face challenges in practical applications. For example, the conformation of DS-Cav1 may gradually change during storage at 4 °C, leading to a decrease in neutralizing antibody antigenicity. In contrast, DT-PreF, despite its better stability, has not yet entered the clinical verification stage [58]. In addition, the current technology has insufficient tolerance to fluctuations in environmental factors such as temperature and pH, which may limit the promotion of vaccines in resource-limited areas.

Future research on RSV vaccines may explore synergistic effects between the PreF and G proteins [70]. While current vaccines focus on the PreF protein, the G protein also plays a key role in viral attachment and immune modulation. ADV110, as one of the few candidate vaccines targeting the G protein, has demonstrated complementary potential to the PreF vaccine by inducing neutralizing antibodies against CCD. In the future, a combined vaccine of PreF and G protein may be explored to cover a wider range of epitopes. Another promising direction involves the development of VLP vaccines. IVX-A12 mimics the natural virus structure through VLP technology and carries both RSV PreF and hMPV antigens. Its multivalent design may enhance the breadth of the immune response. Phase II trials have shown good safety, providing an example for the development of combined vaccines with multiple antigens. Due to the age-related decline in immune function, tailored immunization strategies are crucial for the elderly. These may include developing new vaccination strategies, adjusting the vaccination cycle, or increasing the vaccine dose to overcome the decline of immune function due to aging.

To further optimize vaccine design, it is essential to clarify the immune mechanisms that mediate protection against RSV infection. Neutralization of RSV by antibodies generally refers to in vitro neutralization assays, where antibodies can prevent infection of cell-based assays. This often relies on antibodies blocking cellular entry by blocking RSV binding to specific cellular receptors. In vitro antibody neutralization is independent of the presence of Fc domains since it relies solely on steric hindrance of receptor binding. However, in vivo many other mechanisms occur that help eradicate viruses, which was recently also well-studied for RSV by Bartsch et al. [77]. Using adenovirus-delivered preF vaccine as a model, they showed that protection against RSV infection was linked to various effector functions, including antibody-dependent neutrophil phagocytosis, antibody-dependent complement deposition and antibody-dependent cellular phagocytosis. Furthermore, they showed the importance of IgA responses and differentially glycosylated RSV-specific IgG profiles for protection. Finally, the importance of Fc-mediated effector functions was further demonstrated by improving the potency of a monoclonal antibody by engineering the Fc domain for more efficient binding to Fc-receptors [77]. Assays for Fc-mediated effector functions and cellular immunity are often not employed in RSV vaccine evaluation, and should be included in future studies. This would help avoid misjudgment of vaccine protective efficacy caused by relying solely on in vitro neutralization data.

Global accessibility of RSV vaccines also requires attention. Improving vaccine stability (such as the low-temperature storage advantage of DT-PreF) [58] or developing new preparation technology routes could reduce the dependence on the cold chain, thus promoting the popularization of vaccines in low-and middle-income countries. Concurrently, the development of novel adjuvants represents a key area of innovation, particularly for enhancing the immunogenicity of subunit vaccines in immunocompromised populations. New-generation adjuvants may act through multiple mechanisms. A key mechanism involves enhancing antigen presentation. Adjuvants based on TLR agonist activate dendritic cells (DCs) [78] by upregulating co-stimulatory molecules (e.g., CD80/86) and MHC expression, effectively rejuvenating aged dendritic cells and improving their ability to present antigen to T cells. Complementing this approach, liposomal adjuvants protect antigens from degradation and target them to lymph nodes, compensating for age-related declines in antigen uptake and transport [68]. Beyond antigen presentation, they modulate immune cell function. CpG oligonucleotides promote Th1 responses (e.g., IFN-γ secretion) to counter weakened Th1 activity in the elderly, and alum adjuvants form antigen depots to sustain B cell stimulation, enhancing neutralizing antibody production and addressing reduced B cell function in older individuals [78]. Further contributing to vaccine efficacy, certain adjuvants regulate cytokines and chemokines to optimize the immune environment. Formulations like incomplete Freund’s adjuvant induce chemokines to recruit immune cells (e.g., neutrophils) to injection sites, overcoming age-related migration deficits, while others stimulate IL-12 secretion to promote Th1 differentiation and NK cell activation, optimizing cytokine networks impaired in the elderly.

In summary, while substantial progress has been made in RSV subunit vaccine development—particularly in PreF antigen design—continued innovation is required to address population-specific challenges and to explore novel antigen targets and delivery platforms for broader, more durable protection. To translate these advances into tangible global health benefits, critical considerations of formulation stability, cost-effectiveness, and accessibility in low-and middle-income countries must be integrated in parallel with the pursuit of fundamental research.

## 6. Conclusions

Synergistic advancements across immunology, structural biology, and public health have significantly accelerated RSV vaccine development. Immunology unravels RSV-specific immune mechanisms, informs evaluation tools and provides insights into immune escape. Structural biology determines high-resolution structures of RSV F (prefusion and postfusion conformations) and G proteins, and guides the rational design of stabilized antigens, enhancing vaccine immunogenicity and specificity. Public health quantifies RSV burden, optimizes vaccination strategies and ensures real-world efficacy, safety, and equitable access.

The development of RSV subunit vaccines has been revolutionized by structural insights enabling precise stabilization of the PreF conformation. While current PreF-stabilized antigens represent a major advance, key challenges remain in enhancing thermostability, improving cross-variant protection, and addressing diminished immunogenicity in elderly populations. Future efforts should prioritize structure-guided immunogen design to develop pan-RSV vaccines, explore novel adjuvant combinations to strengthen durable immunity, and optimize age-specific vaccination strategies. Overcoming these hurdles through multidisciplinary collaboration will be crucial to fully realize the public health potential of RSV vaccines.

## Figures and Tables

**Table 1:** Summary of subunit RSV vaccines.

Name	Vaccine Characteristics	Clinical Progress	Target Population	Research Progress	Advantages and Disadvantages
Abrysvo	1. Target protein: RSV PreF 2. Bivalent vaccine, targeting RSV-A and RSV-B subtypes	Available	1. Pregnant women 2. People aged 60 years and older	1. Phase III clinical trial (MATISSE study) showed 81.8% to 69.4% protection against RSV-associated LRTI in infants 2. The ConquerRSV Study has shown a 75% to 82% protection against RSV-related hospitalizations in older adults	Advantages: 1. High safety and efficacy 2. Early protection for babies through maternal immunization Suitable for pregnant women and the elderly Disadvantages: Possibilities of increasing the risk of preterm birth and hypertensive disorders of pregnancy
Arexvy	1. Target protein: RSVPreF3 (pre-fused F protein) 2. Combined with AS01E adjuvants (MPL and QS-21)	Available on the market	1. People aged 60 and above 2. People aged 50 to 59 years at high risk	1. The Phase III clinical trial (AReSVi-006) showed a protective efficacy of 82.6–94.1% against RSV-related lower respiratory tract infection 2. Effectiveness was 67.2–78.8% in both RSV seasons	Advantages: 1. Efficient protection of the elderly population 2. It is suitable for a wide range of people 3. AS01E adjuvant enhances immune response Disadvantages: 1. There may be a risk of GBS. 2. Strict storage conditions
DS-Cav1	1. Target protein: RSV PreF 2. PreF conformation is stabilized by DS and Cav1.	Phase I clinical trial	1. Pregnant women 2. The elderly	1. Phase I clinical trials have shown that it is safe and well tolerated in healthy adults 2. It has long-lasting neutralizing viral activity and may protect multiple RSV epidemic seasons	Advantages: 1. Good tolerability and immunogenicity 2. Potentially protective effect on newborns Disadvantages: 1. Maternal hyperactivity antibodies may have an effect on infant lung development 2. There are insufficient studies in older populations
DT-PreF	1. Target protein: RSV PreF 2. Stabilization of PreF conformation by dityrosine bonds 3. High temperature stability	It has not yet entered clinical trials	1. The elderly 2. People with weakened immune systems	1. Animal experiments have shown a strong protective effect 2. High doses overcome immunosenescence without the need for adjuvants or repeated dosing	Advantages: 1. Stronger low-temperature stability 2. Efficient neutralizing antibody titers 3. Immunosenescence can be overcome by increasing the dose Disadvantages: It has not entered clinical trials
ADV110	1. Target protein: RSV G protein CCD 2. Combined with AE011 adjuvant 3. Induce Treg cells to inhibit excessive T cell responses	Completed Phase I and Phase II clinical trials	1. Adults aged 60–80 years	1. Phase II clinical trials showed significant increases in anti-RSV antibody levels 2. It did not induce an excessive T cell response, reducing the risk of VED.	Advantages: 1. High safety and immunogenicity 2. Reduces excessive T cell response and reduces the risk of VED Disadvantages: Treg induction mechanisms may inhibit the antiviral immune response
IVX-A12	1. Target protein: PreF of RSV and hMPV 2. Bivalent vaccine based on VLP technology	Phase II clinical trial	1. Seniors aged 60–75 years	1. Phase II clinical trials showed a robust immune response and a favorable safety profile 2. Phase III trial is about to begin	Advantages: 1. Multivalent design enhances immune response 2. Good safety and application prospects Disadvantages: Phase III trials have not yet been completed
